# Yeast peptides alleviate lipopolysaccharide-induced intestinal barrier damage in rabbits involving Toll-like receptor signaling pathway modulation and gut microbiota regulation

**DOI:** 10.3389/fvets.2024.1393434

**Published:** 2024-06-26

**Authors:** Jiaqi Fan, Chong Li, Wenxiao Han, Fengyang Wu, Huimin Fan, Dongfeng Fan, Yajuan Liu, Zilin Gu, Yuanyuan Wang, Saijuan Chen, Baojiang Chen

**Affiliations:** ^1^College of Animal Science and Technology, Hebei Agricultural University, Baoding, China; ^2^Mountainous Area Research Institute of Hebei Province, Hebei Agricultural University, Baoding, China; ^3^Agricultural Technology Innovation Center in Mountainous Areas of Hebei Province, Baoding, China; ^4^Agricultural Comprehensive Management Detachment of Tangshan City, Tangshan, China

**Keywords:** yeast peptides, rabbit, lipopolysaccharide, intestinal barrier, cecal microbiota, intestinal tight junction, inflammatory

## Abstract

**Introduction:**

Yeast peptides have garnered attention as valuable nutritional modifiers due to their potential health benefits. However, the precise mechanisms underlying their effects remain elusive. This study aims to explore the potential of yeast peptides, when added to diets, to mitigate lipopolysaccharide (LPS)-induced intestinal damage and microbiota alterations in rabbits.

**Methods:**

A total of 160 35-day-old Hyla line rabbits (0.96 ± 0.06 kg) were randomly assigned to 4 groups. These groups constituted a 2 × 2 factorial arrangement: basal diet (CON), 100 mg/kg yeast peptide diet (YP), LPS challenge + basal diet (LPS), LPS challenge +100 mg/kg yeast peptide diet (L-YP). The experiment spanned 35 days, encompassing a 7-day pre-feeding period and a 28-day formal trial.

**Results:**

The results indicated that yeast peptides mitigated the intestinal barrier damage induced by LPS, as evidenced by a significant reduction in serum Diamine oxidase and D-lactic acid levels in rabbits in the L-YP group compared to the LPS group (*p* < 0.05). Furthermore, in the jejunum, the L-YP group exhibited a significantly higher villus height compared to the LPS group (*p* < 0.05). In comparison to the LPS group, the L-YP rabbits significantly upregulated the expression of *Claudin-1*, *Occludin-1* and *ZO-1* in the jejunum (*p* < 0.05). Compared with the CON group, the YP group significantly reduced the levels of rabbit jejunal inflammatory cytokines (TNF-α, IL-1β and IL-6) and decreased the relative mRNA expression of jejunal signaling pathway-associated inflammatory factors such as *TLR4*, *MyD88*, *NF-κB* and *IL-1β* (*p* < 0.05). Additionally, notable changes in the hindgut also included the concentration of short-chain fatty acids (SCFA) of the YP group was significantly higher than that of the CON group (*p* < 0.05). 16S RNA sequencing revealed a substantial impact of yeast peptides on the composition of the cecal microbiota. Correlation analyses indicated potential associations of specific gut microbiota with jejunal inflammatory factors, tight junction proteins, and SCFA.

**Conclusion:**

In conclusion, yeast peptides have shown promise in mitigating LPS-induced intestinal barrier damage in rabbits through their anti-inflammatory effects, modulation of the gut microbiota, and maintenance of intestinal tight junctions.

## Introduction

Rabbit meat is indeed renowned for its delectable taste and nutritional richness, making it a beloved choice among consumers. However, during the weaning period, the gastrointestinal tract of rabbits is not fully developed, and at the same time, it is subjected to the stress of cage transfer and feed change, which is very easy to be attacked by bacteria, causing gastrointestinal diseases, and then affecting the healthy growth of rabbits (1).

The gastrointestinal tract plays a vital role in maintaining the health of both humans and animals, serving not only for the digestion and absorption of nutrients but also as a protective barrier against bacterial invasion and toxin entry into the internal environment. Intestinal damage increases the permeability of the intestinal barrier, allowing pathogens and toxins to enter the internal environment from the intestinal lumen. This exacerbates both intestinal and systemic inflammation (2). Previous studies have shown that the nuclear-factor kappa B (NF-kB) pathway is associated with intestinal barrier function, and inhibition of NF-kB pathway can reduce intestinal inflammation and improve intestinal epithelial dysfunction (3). Therefore, regulation of NF-kB pathways by nutritional supplements in the gastrointestinal tract, followed by down-regulation of pro-inflammatory factor genes, may have beneficial effects in alleviating intestinal injury. Moreover, the intestinal microbiota plays a crucial role in maintaining host health. A stable intestinal microbiota promotes the upkeep of the intestinal mucosal barrier and modulates the immune defense system (4, 5). During inflammation, an altered Firmicutes-to-Proteobacteria ratio characterizes the flora imbalance, with an increased abundance of the latter, containing pathogenic bacteria, and a decrease in beneficial bacteria (e.g., *Faecalibacterium*), thereby weakening the intestinal function (6). Optimized gut microbes can produce short-chain fatty acids (SCFA) through the fermentation of dietary fiber, participating in crucial regulatory and metabolic processes, including the inhibition of intestinal inflammation (7). As a result, nutritional regulation of the intestinal microbiome is an effective method to improve intestinal health.

Yeast peptides, originating from induced *Saccharomyces cerevisiae*, are antimicrobial peptides with diverse biological activities, encompassing anti-inflammatory, anti-stress, growth promotion, and immunomodulation (8–10). These peptides are produced microbially and have found extensive application in livestock and poultry farming, where they modulate epithelial function to exert anti-inflammatory effects on the intestinal barrier (11, 12). Various studies have demonstrated that peptides from *Enterobacteriaceae* and synthetic antimicrobial peptides can significantly reduce serum and intestinal levels of inflammatory factors such as TNF-α, IL-1, and IL-6 in piglets, highlighting potent anti-inflammatory effects (13). Cathelicidin antimicrobial peptides were observed to mitigate LPS-induced elevation of pro-inflammatory cytokine expression in the intestinal tract of mice, thereby enhancing the intestinal barrier and constraining inflammatory responses (14). Additionally, the antimicrobial peptide MccJ25 was identified to alleviate intestinal inflammation and enhance the function of the disrupted intestinal barrier in a mouse model of ulcerative colitis (UC) by modulating the balance of Lactobacillus, Bacteroides, and Akkermansia. Therefore, we suspect that yeast peptides may play a crucial role in preventing intestinal diseases in rabbits.

However, the impact of yeast peptides on the gut development and microbiota following LPS attack remains unexplored. This study aims to investigate the influence of yeast peptides on gut function and cecum microbiota in LPS-infected rabbits. Our investigation encompasses an examination of gut morphology, mRNA expression of intestinal tight junction proteins, and inflammatory cytokines. Furthermore, we explore alterations in the cecum microbial community subsequent to yeast peptide supplementation and LPS attack, offering insights into potential mechanisms underlying intestinal damage and repair.

## Materials and methods

### Experimental design, animal and management

One hundred and sixty healthy weaned rabbits (Hyla line rabbits, 35-day-old, initial body weight of 0.96 ± 0.06 kg, equally distributed by gender) were randomly allocated to 4 groups with eight replicates of five rabbits each. The groups followed a completely randomized 2 × 2 factorial arrangement, with dietary treatments (basal diet or 100 mg/kg of yeast peptide) and LPS treatments (saline or LPS injection) as the primary factors. The groups were defined as follows: (1) CON group: basal diet with thigh muscle injection of saline, (2) YP group: basal diet supplemented with 100 mg/kg yeast peptide, (3) LPS group: basal diet with thigh muscle injection of LPS, and (4) L-YP group: basal diet supplemented with 100 mg/kg YP and thigh muscle injection of LPS. The main components of yeast peptide are: yeast peptide ≥5,000 mg/kg, crude protein ≥3%, mannan ≥0.5%, crude ash ≤11%, water ≤11%. Purchased from Beijing Enhalor Biotechnology Co., Ltd., it has a molecular weight of 1.9 kD and consists of 19 amino acid residues (GGVGKIIEYFIGGGVGRYG). The basal diet adhered to the nutritional requirements for rabbits recommended by the National Research Council (15), and its composition and nutritional levels are detailed in Table 1.

**Table 1:** Composition and nutrient levels of the basal diet (air-dry basis, %).

Ingredients	Content	Nutritional level^b^	Content
Corn	9.00	Digestible energy (MJ/kg)	10.15
Wheat bran	20.00	Crude protein	16.79
Soybean meal	2.00	Ether extract	3.27
Wheat middling	8.00	Crude fiber	15.32
Sunflower cake	11.00	Crude ash	11.27
malt root	12.00	Neutral detergent fiber	41.56
Peanut hulls	22.00	Acid detergent fiber	24.83
*Artemsia argyi* powder	5.00	Acid detergent lignin	5.71
Limestone	1.50	Calcium	1.34
NaCl	0.50	Total phosphorus	0.69
Rice husk powder	7.00		
Ca(HCO_3_)_2_	0.55		
L-lysine hydrochloride	0.35		
DL-Met	0.10		
Premix^a^	1.00		
Total	100.00		

a The premix provided the following per kg of the diet: Fe 70 mg, Cu 20 mg, Zn 70 mg, Mn 10 mg, Co 0.15 mg, I 0.2 mg, Se 0.25 mg, VE 50 mg, VK 2 mg, VB1 2 mg. VB2 6 mg, VB5 50 mg, VB6 2 mg, VB12 0.02 mg, VB3 50 mg, nicotinic acid niacin 20 mg, pantothenic acid 12.5 mg, VA 10,000 IU, VD 900 IU, choline 1,000 mg, biotin 0.2 mg.

b Digestible energy was a calculated value, while the others were measured values.

The feeding trial spanned a total of 35 days, comprising a 7-day pre-test and a 28-day formal test period. Rabbits were individually housed in cages (L × W × H: 120 cm × 80 cm × 60 cm), provided *ad libitum* access to feed and water throughout the experimental duration, feeding a fixed amount of feed twice a day (once at 8:00, and once at 16:00) and immunized following established procedures. On the 28th day of the experiment, one rabbit was randomly selected from each replicate. Consistent with our laboratory’s prior reports, the LPS and L-YP groups received a thigh muscle injection of 1 mL *Escherichia coli* LPS (*Escherichia coli* O55: B5, L2880, Sigma-Aldrich) at a dose of 200 μg/kg body weight, while the CON and YP groups received an equivalent amount of physiological saline.

### Samples collection

On day 28 of the formal test period, one rabbit per replicate was selected, and blood was collected 4 h after injection of LPS or saline. The blood, obtained by venipuncture at the ear margin, was collected in 5 mL volumes, subjected to centrifugation at 4°C and 3,000 × g for 10 min, and the resulting supernatant was collected and stored frozen. Following blood collection, rabbits were euthanized with sodium pentobarbital [100 mg/kg IV, guidelines from the American Veterinary Medical Association (AVMA)]. A 3 cm section of duodenum, jejunum, ileum, and cecum tissue was promptly isolated without any treatment.

For intestinal histology, segments of the jejunum and cecum were gently rinsed three times with ice-cold saline, sheared into 1 cm segments, and then fixed in a 4% paraformaldehyde solution. Additionally, jejunal and cecal samples were collected in sterile sampling tubes and stored at −80°C for subsequent analysis.

### Determination of diamine oxidase and D-lactic acid levels in serum

Samples were tested using ELISA kits (acquired from Shanghai Enzyme-linked Biotechnology Co., Ltd.). Serum samples were taken, 10 μL of the sample to be tested was added to the sample well, then 40 μL of the sample diluent was added, and 100 μL of HRP was added to the sample well. The reaction hole was sealed with sealing plate film and incubated at 37°C for 60 min. Then discard the liquid and wash it 5 times with detergent. 50 μL of substrate A and B were added to each well and incubated at 37°C for 15 min without light. Finally, 50 μL of the termination solution was added and measured the OD value of each hole at a wavelength of 450 nm in 15 min. In the Excel worksheet, the standard product concentration is taken as the horizontal coordinate and the corresponding OD value is taken as the vertical coordinate to draw the linear regression curve of the standard product. The concentration range of the DAO calibration curve: 0–24 U/mL. The concentration range of the DLA calibration curve: 0–80 nmol/L. Concentrations of D-lactic acid (DLA, Cat. No. ml974725) and Diamine oxidase (DAO, Cat. No. ml027888) in serum were calculated by curve equation.

### Intestinal morphology

Jejunum and cecum samples were immersed in a 4% paraformaldehyde solution for 48 h to facilitate intestinal histological observation. Subsequently, these samples underwent dehydration using a JT-12S automatic dehydrator (Junjie Electronics Co., Ltd., Wuhan, China), embedding in a BMJ-A tissue embedding machine (Zhongwei Electronic Instrument Factory, Changzhou, China), and slicing into 5 μm thin sections using a rotary slicer (Leica Biosystems, Wetzla, Germany). Staining was performed with an RS36 staining machine (P.S.J. Medical Devices Co., Ltd., Changzhou, China), followed by sealing. Digital trinocular images were acquired using a BA210Digital microscope (McAuldie Industrial Group Co., Ltd.) with 40× magnification selected for observation. The height of villi and the depth of crypts were measured using the image analysis software Motic Images Advanced 3.2, and the ratio of villi height to crypt depth was calculated. Each section underwent 10 measurements, and the results were averaged.

### Intestinal inflammatory factor

Samples were tested using ELISA kits (acquired from Shanghai Enzyme-linked Biotechnology Co., Ltd.). The jejunal tissue was ground into tissue homogenate at 4°C in an ice bath to make the sample to be tested. Ten microliters of the sample to be tested was added to the sample well, then 40 μL of the sample diluent was added, and 100 μL of HRP was added to the sample well. The reaction hole was sealed with sealing plate film and incubated at 37°C for 60 min. Then discard the liquid and wash it 5 times with detergent. Subsequently, 50 μL of substrate A and B were added to each well and incubated at 37°C for 15 min without light. Finally, 50 μL of the termination solution was added and measured the OD value of each hole at a wavelength of 450 nm in 15 min. In the Excel worksheet, the standard product concentration is taken as the horizontal coordinate and the corresponding OD value is taken as the vertical coordinate to draw the linear regression curve of the standard product. The concentration range of the TNF-α calibration curve: 0–320 pg/mL. The concentration range of the INF-γ calibration curve: 0–800 pg/mL. The concentration range of the SIgA calibration curve: 0–20 μg/mL. The concentration range of the IL-1β calibration curve: 0–80 pg./mL. The concentration range of the IL-6 calibration curve: 0–120 pg/mL. The concentration range of the IL-10 calibration curve: 0–64 pg/mL. Concentrations of tumor necrosis factor-alpha (TNF-α, Cat. No. ml028087), interferon-gamma (INF-γ, Cat. No. ml041640), Simplified improved Gaussian approximation (SIgA, Cat. No. ml036798), interleukin-1 beta (IL-1β, Cat. No. ml027836), interleukin-6 (IL-6, Cat. No. ml027844) and interleukin-10 (IL-10, Cat. No. ml027828) in jejunum were calculated by curve equation.

### Short-chain fatty acids

A 1 g sample of cecum was weighed in a 5 mL centrifuge tube, followed by the addition of 3 mL of ultrapure water. Thorough mixing was achieved using a vortex mixer, and subsequent centrifugation at 4°C, 12,000 × g for 10 min was performed. The resulting supernatant was collected, and 1/5 volume of the supernatant was combined with 25% metaphosphoric acid. This new solution underwent an additional centrifugation at 12,000 × g for 10 min before being filtered into a chromatographic injection vial using a 0.45 mm filter membrane for the determination of short-chain fatty acids (SCFA).

Detection was carried out using a gas chromatograph (GC-2014, Shimadzu, Japan). The chromatographic column dimensions were 30 m × 0.5 μm × 0.32 mm, with nitrogen serving as the carrier gas at a flow rate of 85.1 mL/min. The temperatures of the column, detector, and injector were set at 120°C, 250°C, and 220°C, respectively.

### qRT-PCR

The relative mRNA expression levels of genes including Toll-like receptors 4 (TLR4), Myeloid differentiation factor 88 (MyD88), Inhibitor of nuclear factor kappaB (IκBα), Nuclear factor kappa-B (NF-κB), TNF-α, IL-1β, IL-6, IL-10, ZO-1, Occludin, and Claudin-1 in the jejunum were determined using real-time fluorescence quantification. Primers for these genes are detailed in Table 2. Total RNA extraction from jejunum was carried out using Trizol reagent (Invitrogen, Carlsbad, United States), and the concentration of the extracted RNA was measured using a Nanodrop Lite RNA concentration needle (Thermo Fisher Scientific, Massachusetts, United States). Subsequent cDNA synthesis utilized the PrimeScript RT Reagent kit (TaKaRa, Dalian, China) following the manufacturer’s instructions. The real-time fluorescence quantification (qRT-PCR) was conducted on an MA-6000 instrument (Shanghai, China), with a reaction system volume of 20 μL. The qRT-PCR mixture comprised 10 μL of 2 × SYBR real-time PCR premixture, 0.8 μL of forward and reverse primer mixture, 1 μL of cDNA, and 7.2 μL of RNase Free dH_2_O. Thermal cycling conditions involved an initial cycle at 95°C for 5 min, followed by 40 cycles of 95°C for 15 s and 60°C for 30 s. The melting curve analysis concluded the experiment. Repetition of the experiment three times utilized β-actin as the internal reference gene, and the 2^−ΔΔCt^ method was applied to calculate the relative expression of the target gene.


$$\mathrm{Ratio}\left(\mathrm{test}/\mathrm{calibrator}\right)=2^{-\left[\Delta Ct\left(\mathrm{test}\right)-\Delta Ct\left(\mathrm{calibrator}\right)\right]}$$

ΔCt=Ctdesiredgene−Ctreferencegene

ΔΔCt=ΔCtexperimentalgroup−ΔCtcontrolgroup

**Table 2:** Sequence of primers for real-time PCR.

Target gene	Primer sequence (5′–3′)	Product size (bp)	Accession number
TNF-α	Forward: ACCATGGAGTCCTTCAGCTC	144	NC_067386.1
	Reverse: TTTGGACACACGGTAGAGCT		
IL-10	Forward: AAACAAGAGCAAGGCAGTGG	170	NC_067389.1
	Reverse: GGATGGAGTTCTCCTGGCTT		
TLR4	Forward: TGCCACCTGTCAGATAAGCA	121	NC_067374.1
	Reverse: TCGGCCAACTAGAAGCATCA		
MyD88	Forward: AGACCAACTACCGGCTGAAG	171	NW_026259666.1
	Reverse: GGGCAAACTTGGTCTGGAAG		
NF-κB	Forward: CAACCCACCTACCCATCTGT	123	NC_067395.1
	Reverse: GCACACGGGGTTTGAAGAAT		
IL-β	Forward: TGTAGACCCCAACCGTTACC	149	NC_067375.1
	Reverse: GGAAGACGGGCATGTACTCT		
IL-6	Forward: GCAGAACCATCGAGAGCATC	178	NC_067383.1
	Reverse: GTCTCATTATTCACCGCCGG		
IkBα	Forward: TGCACTTGGCCATCATCCAT	190	NM_020529.3
	Reverse: TCTCGGAGCTCAGGATCACA		
Occludin	Forward: ATTTTGACACTGGCCTGCAG	143	NC_067384.1
	Reverse: GTTGTACTCATCAGCAGCCG		
ZO-1	Forward: CCGTGTTGTGGATACCTTG	89	NC_067390.1
	Reverse: TGCCTCGTTCTACCTCCTT		
Claudins-1	Forward: AGCATGGTATGGCAACAGA	121	NC_067387.1
	Reverse: CAGAAGGCAGAGAGAAGCA		
β-actin	Forward: CCTGGCACCCAGCACAAT	118	NW_026259299.1
	Reverse: GCTGATCCACATCTGCTGGAA		

### Cecum microflora

The total genomic DNA of the microbial community was extracted following the guidelines of the E.Z.N.A.^®^ soil DNA kit (Omega Biotek, Norcross, GA, United States). The extracted genomic DNA’s quality was assessed through 1% agarose gel electrophoresis, and DNA concentration and purity were determined using NanoDrop 2000 (Thermo Scientific, United States). The DNA served as a template for PCR amplification of the V3–V4 variable region of the 16S rRNA gene, employing the primers 338F (5′-ACTCCTACGGG GAGGCAGCAG-3′) and 806R (5′-GGACTACHVGGGTWTCT AAT-3′) containing the Barcode sequence. The PCR reaction mixture comprised 4 μL of 5× TransStart FastPfu buffer, 2 μL of 2.5 mM dNTPs, 0.8 μL of each 5 μM primer, 0.4 μL of TransStart FastPfu DNA polymerase, and 10 ng of template DNA, adjusted to a final volume of 20 μL. The amplification process consisted of an initial pre-denaturation at 95°C for 3 min, followed by 27 cycles of denaturation at 95°C for 30 s, annealing at 55°C for 30 s, and extension at 72°C for 30 s, concluding with a final stabilization and extension at 72°C for 10 min. The samples were stored at 4°C (PCR instrument: ABI GeneAmp^®^ 9700). The resulting products underwent purification using the AxyPrep DNA Gel Extraction Kit (Axygen Biosciences, Union City, CA, United States), were verified by 2% agarose gel electrophoresis, and quantified with the Quantus^™^ Fluorometer (Promega, United States). The purified PCR products were then utilized for library construction with the NEXTFLEX Rapid DNA-Seq Kit and subsequent sequencing on Illumina’s Miseq PE300 platform (Shanghai Meiji Biomedical Technology Co., Ltd.).

### Statistical analysis

ANOVA test was performed for statistical analysis, and general linear model procedures of SPSS 26.0 for a 2 × 2 factorial design were used (IBM SPSS Inc., Chicago, Illinois, United States). Each rabbit served as the statistical unit (*n* = 8). Serum and jejunum data were checked for normal distribution using the Shapiro Wilk test. Statistical models encompassed daily supplementation with yeast peptides (0 or 100 mg/kg), LPS treatments (saline or LPS injection) and their interactions. Statistical model: *y_ijk_* = *μ* + *α_i_* + *β_j_* + *α_i_* × *β_j_* + *e_ijk_* where *y_ijk_* is an observed trait, *μ* is the overall mean, *α_i_* is the fixed effect of immunological challenge (*i* = saline or LPS), *β_j_* is the fixed effect of dietary YP (*j* = 0 or 100 mg/kg YP), *α_i_* × *β_j_* is the interaction between LPS and YP and *e_ijk_* is the random error. When there was a significant trend for interaction, data were further analyzed by use of one-way ANOVA with Duncan’s multiple range tests. A treatment difference achieved significance at *p <* 0.05, while a trend was denoted when 0.05 < *p <* 0.10. Results were reported as mean ± standard error of the mean (SEM). The calculation of the Alpha diversity index followed, with the Wilcoxon rank sum test applied to analyze between-group differences in Alpha diversity. Principal Coordinate Analysis (PCoA) based on the Bray–Curtis distance algorithm tested the beta diversity of microbial communities among the samples. The non-parametric PERMANOVA test assessed the significance of differences in microbial community structure between sample groups. Additionally, Linear Discriminant Analysis Effect Size (LEfSe) analysis (LDA >2, *p* < 0.05) was utilized to identify bacterial taxa exhibiting significant differences in abundance at the genus level across distinct groups. Correlations between bacterial abundance (at the genera level) and environmental factors were evaluated by Spearman’s correlation test by use of the R language package “Pheatmap.”

## Results

### Effect of YP on serum levels of DAO and DLA in LPS-induced rabbits

**Figure 1:**
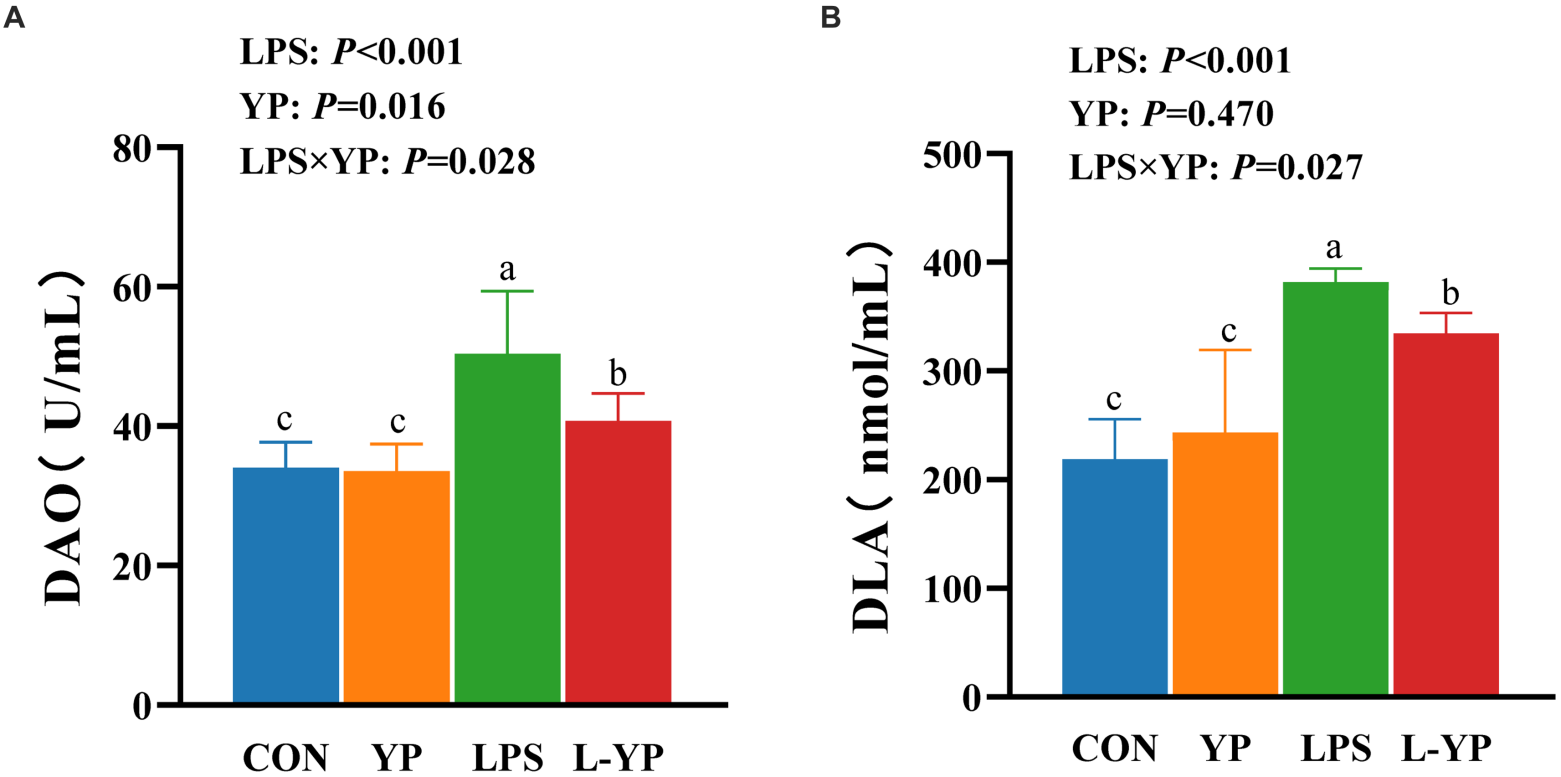
Effect of dietary yeast peptides on **(A)** serum DAO and **(B)** DLA concentration in LPS-induced rabbits. DAO, diamine oxidase; DLA, D-lactic acid; CON, rabbits fed with the basal diet; YP, rabbits fed with the basal diet and 100 mg/kg yeast peptides; LPS, rabbits challenged by LPS and fed with the basal diet; L-YP, rabbits challenged by LPS and fed with the basal diet and 100 mg/kg yeast peptides. Vertical bars represent the SEM. ^a–c^Means with no common superscript within each row are significantly (*p* < 0.05, *n* = 8) different.

As depicted in Figure 1, compared with the CON group, LPS challenge significantly elevated serum DAO activity (*p <* 0.05) and DLA content (*p <* 0.05) (Figures 1A,B). There were no significant differences in DAO and DLA levels in the YP group compared to the CON group (*p >* 0.05). Notably, a significant interaction between LPS and yeast peptide was observed for both serum DAO activity and DLA content (*p* < 0.05). Compared with LPS group, serum DAO activity and DLA content in L-YP group were decreased by 19.11 and 12.36%.

### Effect of YP on intestinal morphology in LPS-induced rabbits

**Figure 2:**
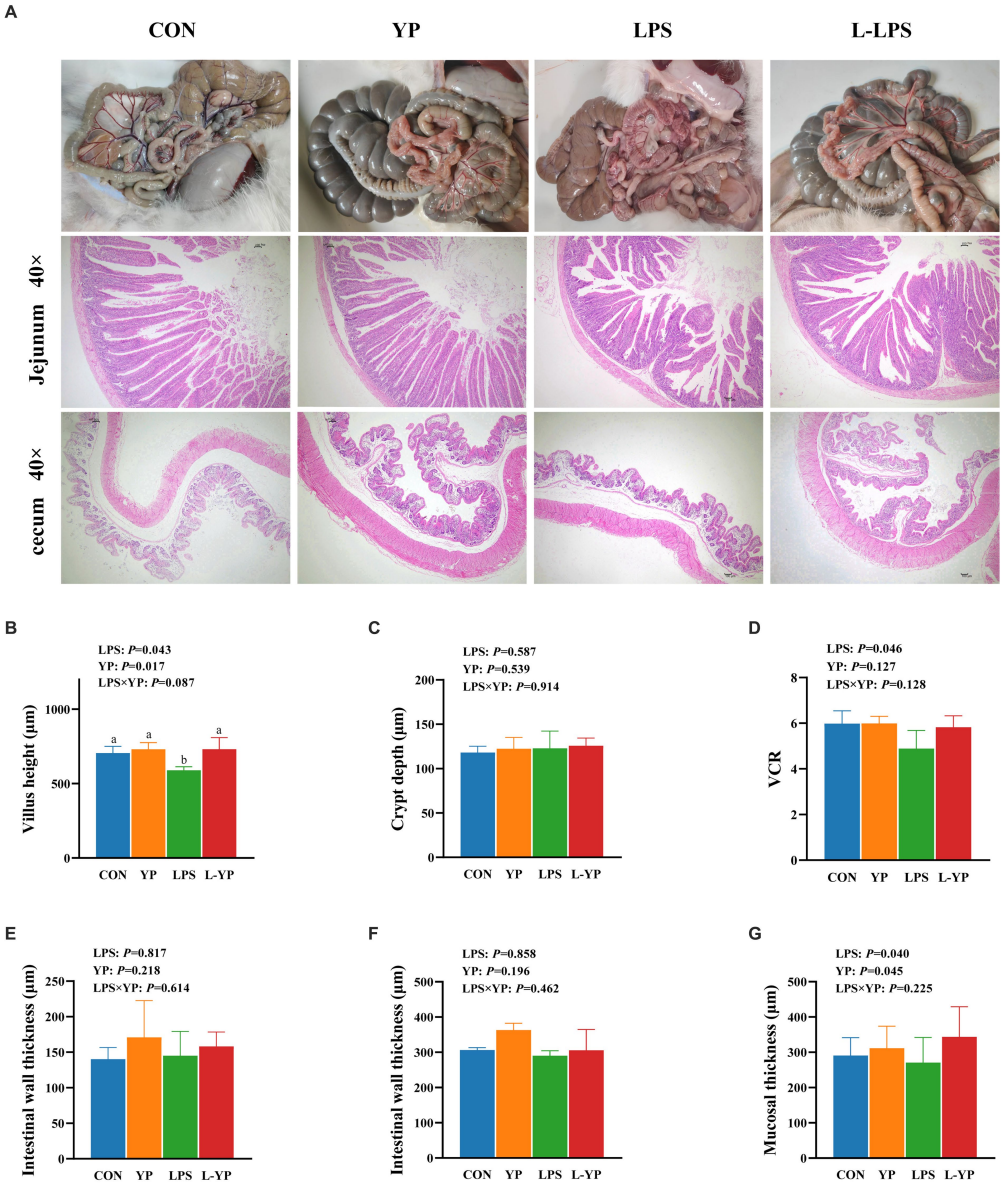
Effect of dietary yeast peptides on intestinal morphology in LPS-induced rabbits. **(A)** Representative picture of the appearance of the intestinal tract and section of jejunum of a rabbit. **(B–E)** Jejunum morphology. **(F,G)** Cecum morphologically. VCR, ratio of villus height to crypt depth; CON, rabbits fed with the basal diet; YP, rabbits fed with the basal diet and 100 mg/kg yeast peptides; LPS, rabbits challenged by LPS and fed with the basal diet; L-YP, rabbits challenged by LPS and fed with the basal diet and 100 mg/kg yeast peptides. Vertical bars represent the SEM. ^a–c^Means with no common superscript within each row are significantly (*p* < 0.05, *n* = 8) different.

Figure 2 illustrates the impact of yeast peptides and LPS stress on the intestinal morphology of rabbit. LPS challenge induced pronounced intestinal congestion (Figure 2A), leading to mucosal damage characterized by atrophied small intestinal villi and mucosal detachment (Figure 2B). In comparison to the CON group, LPS challenge resulted in significant intestinal mucosal injury, marked by a reduction in cecal mucosal thickness (*p <* 0.05), a notable decrease in jejunal villus height and the VCR (*p* < 0.05). However, no significant changes were observed in the thickness of the intestinal wall or the depth of the crypts (*p* > 0.05). Simultaneously, the mucosal depth of the jejunum exhibited a significant reduction (*p* < 0.05).

There was no significant interaction between yeast peptides and LPS stress on intestinal morphology (*p* > 0.05). Nevertheless, yeast peptides demonstrated a beneficial effect by enhancing the height of jejunal villi in LPS-stressed jejunum (*p* < 0.10). Compared with the control group, yeast peptides had no significant effects on jejunum villus height (*p* > 0.05) and cecal mucosal depth (*p* > 0.05).

### Effect of YP on mRNA expression of jejunal tight junction protein in LPS-induced rabbits

**Figure 3:**
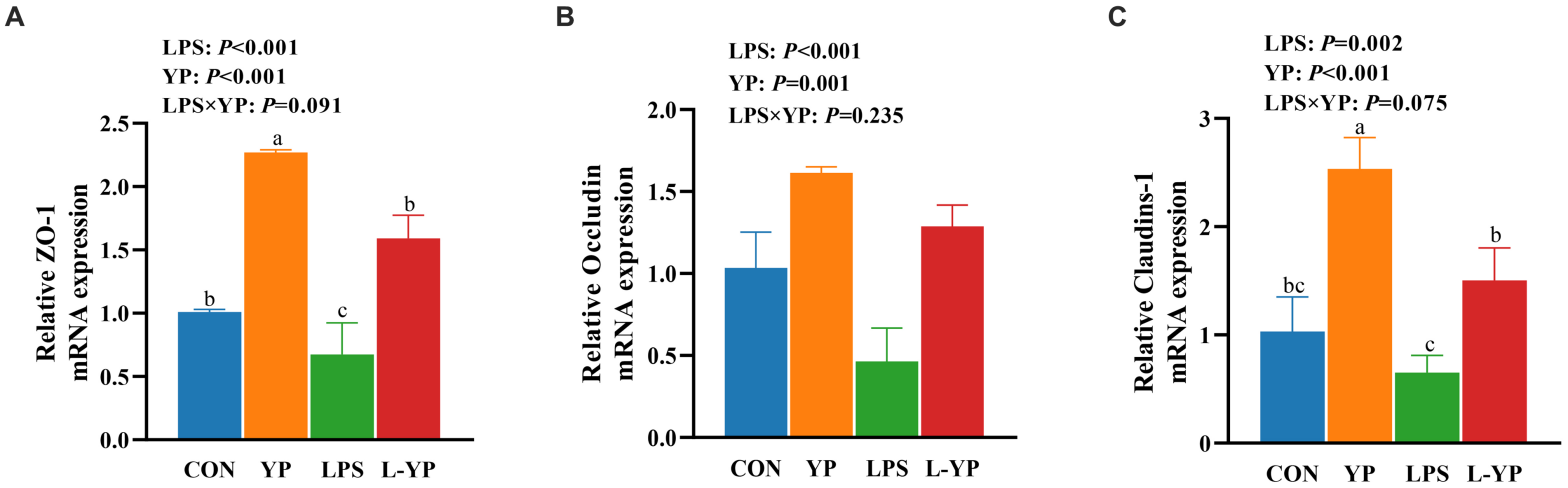
Effects of yeast peptides on the mRNA levels of intestinal tight junction protein of jejunum in LPS-induced rabbits. The mRNA expression of **(A)** ZO-1, **(B)** occludin, and **(C)** claudins-1 was quantitated by real-time PCR. ZO-1, zonula occludens-1; CON, rabbits fed with the basal diet; YP, rabbits fed with the basal diet and 100 mg/kg yeast peptides; LPS, rabbits challenged by LPS and fed with the basal diet; L-YP, rabbits challenged by LPS and fed with the basal diet and 100 mg/kg yeast peptides. Vertical bars represent the SEM. ^a–c^Means with no common superscript within each row are significantly (*p* < 0.05, *n* = 5) different.

As depicted in Figure 3, LPS stress resulted in a notable decrease in the relative expression of Occludin, ZO-1, and Claudin-1 mRNAs compared to the control group (*p* < 0.05). Conversely, yeast peptides exhibited a significant augmentation in the relative expression of Occludin, ZO-1 and Claudin-1 mRNAs (*p* < 0.05). Notably, yeast peptides demonstrated a tendency to increase the mRNA expression of ZO-1 and Claudin-1 compared to the LPS group (*p* < 0.10).

### Effect of YP on inflammatory factor in LPS-induced rabbits

**Figure 4:**
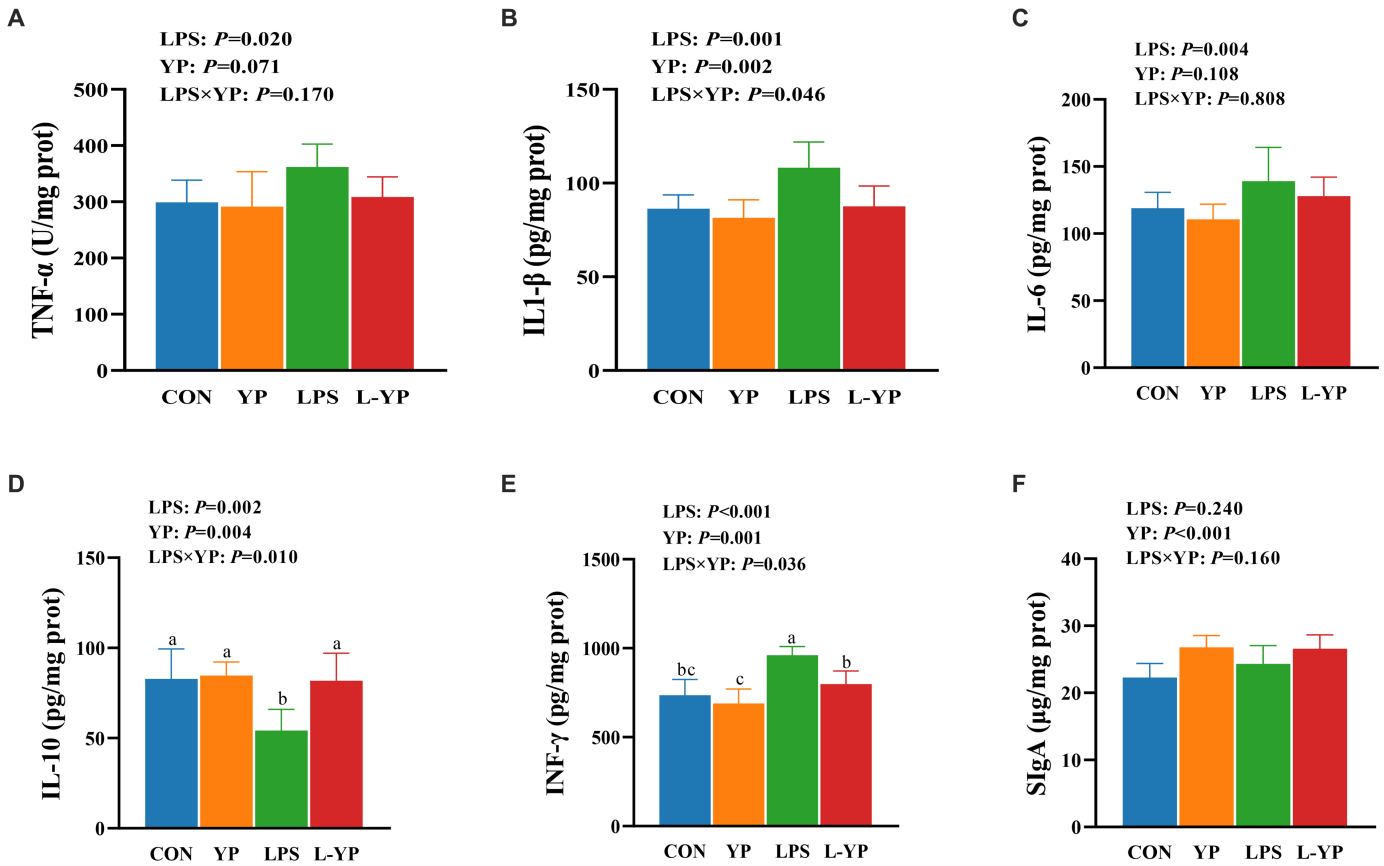
Effects of yeast peptides on the inflammatory factor in LPS-induced rabbits. The contents of **(A)** TNF-α, **(B)** IL-1β, **(C)** IL-6, **(D)** IL-10, **(E)** IFN-γ and **(F)** SIgA in jejunum tissues were determined by ELISA kit. TNF-α, tumor necrosis factor-alpha; IL-1β, interleukin-1 beta; IL-6, interleukin-6; IL-10, interleukin-10; IFN-γ, interferon-gamma; SIgA, secretory immunoglobulin A; CON, rabbits fed with the basal diet; YP, rabbits fed with the basal diet and 100 mg/kg yeast peptides; LPS, rabbits challenged by LPS and fed with the basal diet; L-YP, rabbits challenged by LPS and fed with the basal diet and 100 mg/kg yeast peptides. Vertical bars represent the SEM. ^a–c^Means with no common superscript within each row are significantly (*p* < 0.05, *n* = 8) different.

As illustrated in Figure 4, LPS stress resulted in elevated levels of TNF-α, IL-1β, IL-6 and INF-γ in jejunal tissue (*p* < 0.05), while concurrently reducing levels of IL-10 (*p* < 0.05) compared to controls. There was a significant interaction between yeast peptide and LPS challenge on the contents of IL-1β, IL-10 and INF-γ in jejunal tissue (*p* < 0.05). Notably, yeast peptides demonstrated a positive impact on jejunal tissues by decreasing IL-1β and increasing IL-10 levels (*p* < 0.05), with a tendency to decrease TNF-α (*p* < 0.10).

### Effect of YP on mRNA expression of inflammatory factor in LPS-induced rabbits

**Figure 5:**
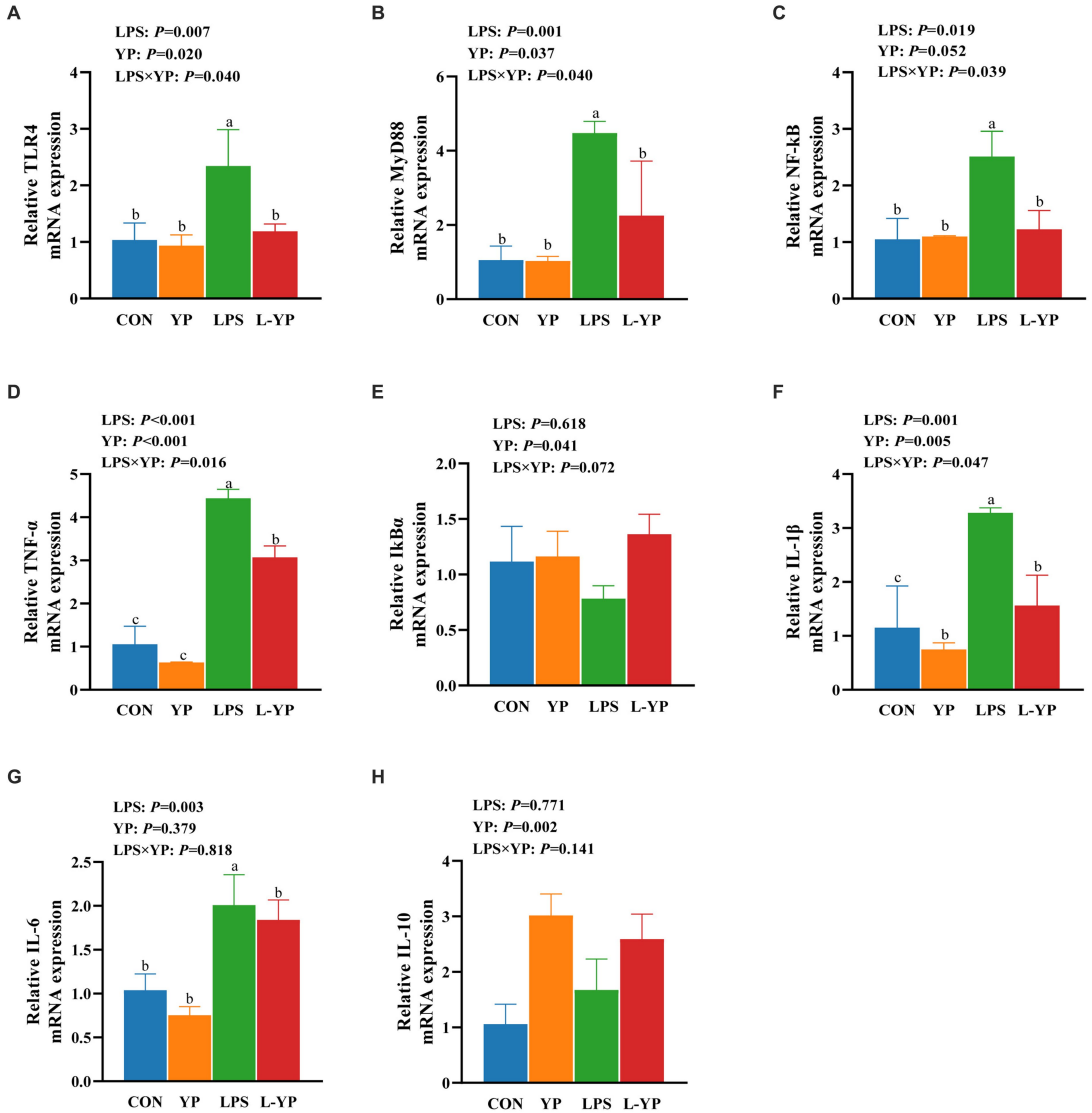
Effects of yeast peptides on the mRNA levels of inflammatory factor of jejunum in LPS-induced rabbits. The mRNA expression of **(A)** TLR4, **(B)** MYD88, **(C)** NF-κB, **(D)** TNF-α, **(E)** IκBα, **(F)** IL-1β, **(G)** IL-6 and **(H)** IL-10 was quantitated by real-time PCR. TLR4, Toll-like receptor 4; MYD88, myeloid differentiation factor 88; NF-κB, nuclear factor kappa-B; TNF-α, tumor necrosis factor-alpha; IL-1β, interleukin-1 beta; IκBα, IkappaB alpha; IL-6, interleukin-6; IL-10, interleukin-10; CON, rabbits fed with the basal diet; YP, rabbits fed with the basal diet and 100 mg/kg yeast peptides; LPS, rabbits challenged by LPS and fed with the basal diet; L-YP, rabbits challenged by LPS and fed with the basal diet and 100 mg/kg yeast peptides. Vertical bars represent the SEM. ^a–c^Means with no common superscript within each row are significantly (*p* < 0.05, *n* = 5) different.

As depicted in Figure 5, LPS stress resulted in a significant elevation of mRNA expression levels for *TLR4*, *MyD88*, *NF-kB*, *TNF-*α, *IL-1β* and *IL-6* in jejunal tissue compared to the control group (*p* < 0.05), the mRNA expression level of *IkBα* was decreased. Compared with the control group, the yeast peptide significantly reduced the mRNA expression of *IL-1β* (*p* < 0.05). The interaction between yeast peptides and LPS stress influenced the expression of jejunal inflammatory factors, with yeast peptides partially reversing the effects induced by LPS. In acomparison to the LPS group, the L-YP group exhibited a significant down-regulation in the expression levels of *TLR4*, *MyD88*, *NF-kB*, *TNF-α* and *IL-1β* (*p* < 0.05). Additionally, yeast peptides increased the expression level of *IL-10* mRNA in jejunal tissues compared to the control group (*p* < 0.05).

### Effect of YP on SCFA in LPS-induced rabbits

**Figure 6:**
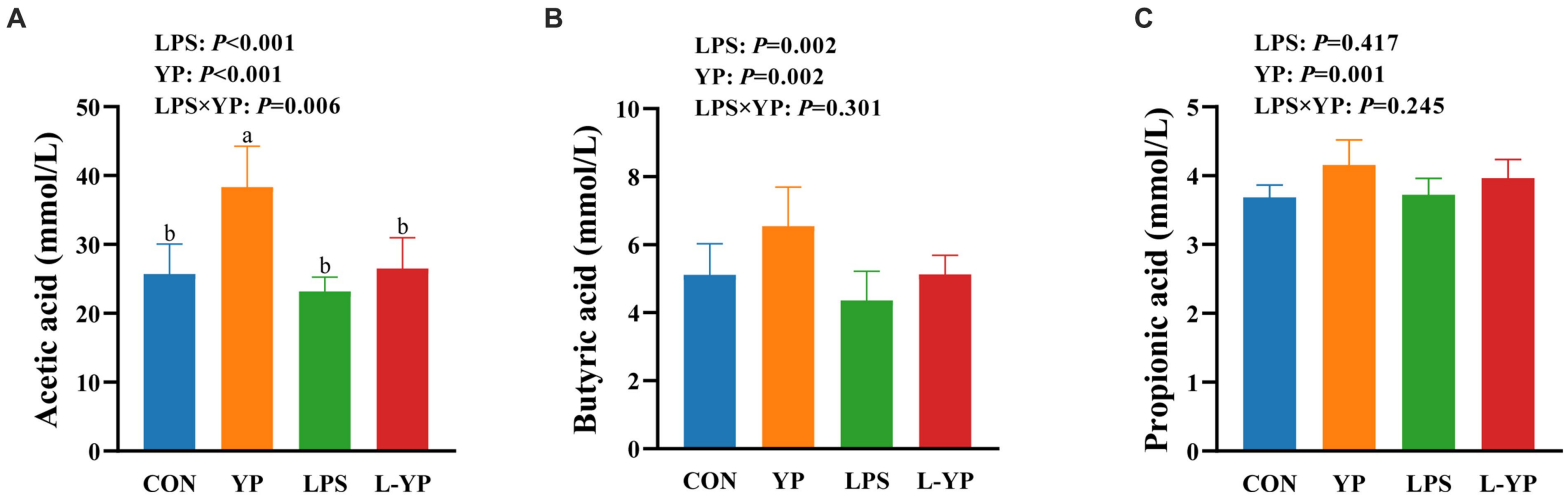
Effects of yeast peptides on the SCFA of cecum digesta in LPS- induced rabbits. The contents of **(A)** acetic acid, **(B)** butyric acid and **(C)** propionic acid were determined by gas chromatograph. CON, rabbits fed with the basal diet; YP, rabbits fed with the basal diet and 100 mg/kg yeast peptides; LPS, rabbits challenged by LPS and fed with the basal diet; L-YP, rabbits challenged by LPS and fed with the basal diet and 100 mg/kg yeast peptides. Vertical bars represent the SEM. ^a–c^Means with no common superscript within each row are significantly (*p* < 0.05, *n* = 8) different.

As depicted in Figure 6, LPS stress resulted in a decrease in the concentrations of acetic acid and propionic acid in the rabbit cecum (*p* < 0.05). There was significant interaction between yeast peptide and LPS challenge on acetic acid concentration (*p* < 0.05). In contrast to the LPS-induced changes, yeast peptides increased the concentrations of acetic, propionic and butyric acids (*p* < 0.05).

### Effect of YP on gut microbial diversity and communities in LPS-induced rabbits

**Figure 7:**
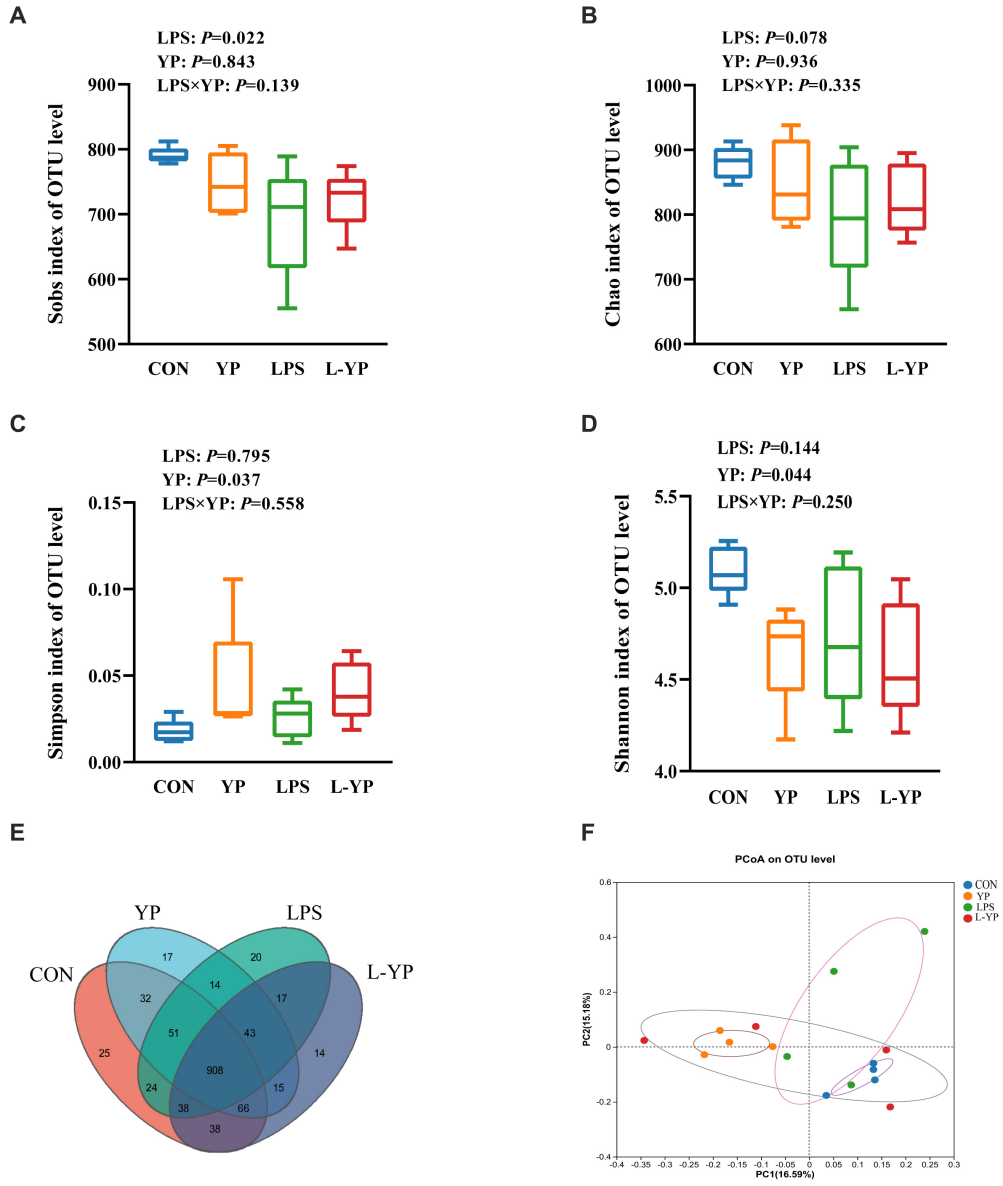
Effects of yeast peptides on the **(A–D)** alpha diversity and **(F)** beta diversity of cecal bacteria in LPS-induced rabbits. Venn graph representation of the shared and exclusive OTU at the 97% similarity level of cecal microbiota **(E)**. CON, rabbits fed with the basal diet; YP, rabbits fed with the basal diet and 100 mg/kg yeast peptides; LPS, rabbits challenged by LPS and fed with the basal diet; L-YP, rabbits challenged by LPS and fed with the basal diet and 100 mg/kg yeast peptides (*n* = 4).

Taxonomic analysis of OTU representative sequences at a 97% similarity level (Figure 7E) yielded a total of 11,826 OTU taxonomic units across all samples. Among these, 908 OTUs were common to all samples, accounting for 7.68% of the total. The number of OTUs unique to each group (CON, YP, LPS, and L-YP) was 25, 17, 20, and 14, respectively. Furthermore, we used Sobs, Chao, Simpson, and Shannon indices to evaluate the impact of yeast peptides and LPS stress on microbial diversity in the cecum of rabbit. As depicted in Figures 7A–D, the Sobs index decreased (*p* < 0.05) in the LPS group, while Simpson’s index increased and Shannon’s index decreased in the YP group (*p* < 0.05), compared to the control group. Additionally, there was a trend of decreasing Chao index in the LPS group (*p* < 0.10). Combined observations indicated no interaction between yeast peptides and LPS stress on microbial α-diversity. To explore similarities or differences in community composition among samples from different subgroups, we conducted Beta diversity through principal coordinates analysis (PCoA) and non-metric multidimensional scaling (NMDS) analysis (Figure 7F). Results demonstrated significant segregation of microbial composition in CON, YP, LPS, and L-YP groups (PCoA-PC1: 16.59% vs. PC2: 15.18%). ANOSIM analysis between groups revealed a significant difference in samples among groups (*p* < 0.05).

**Figure 8:**
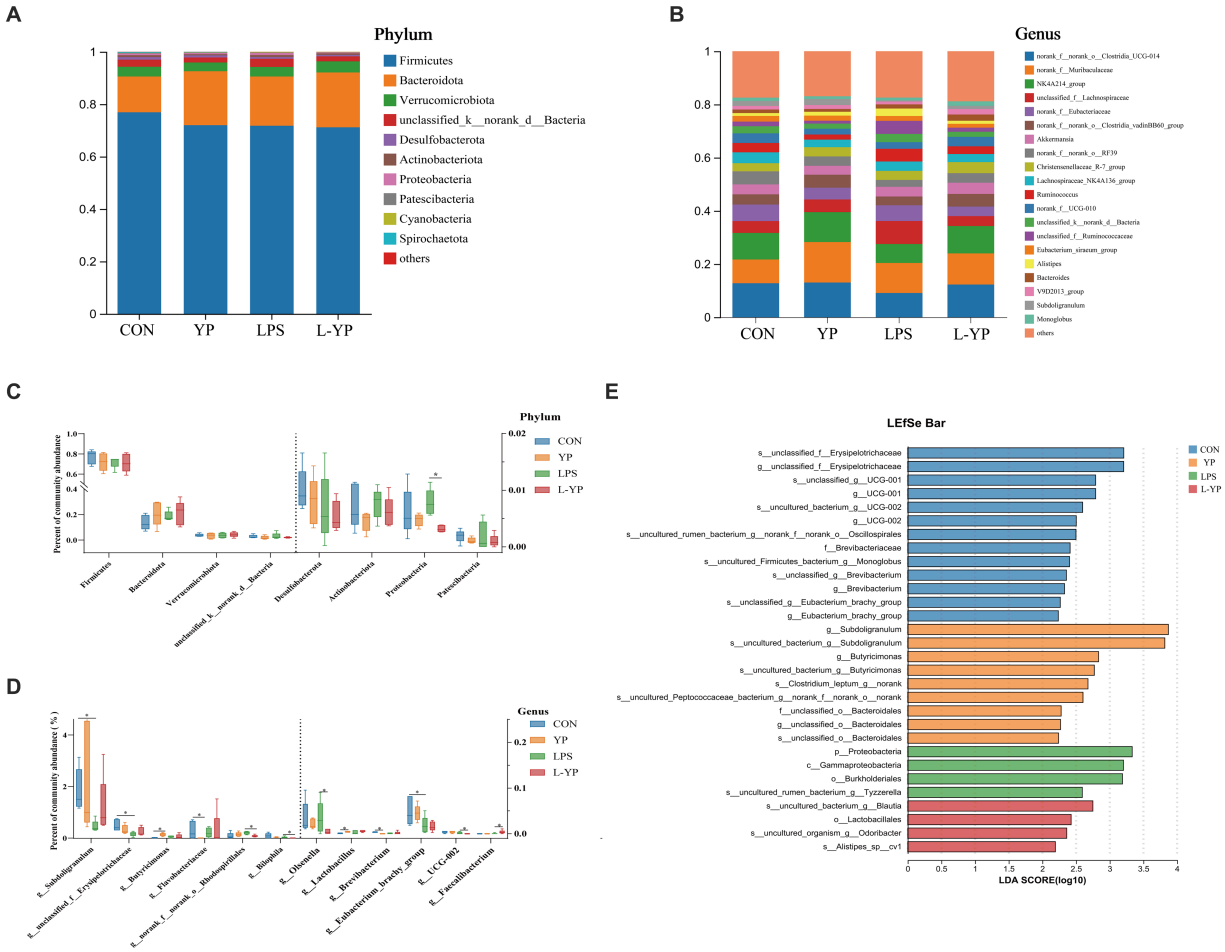
Effects of yeast peptides on the distribution of cecal bacteria at the **(A)** phylum and **(B)** genus levels in LPS-induced rabbits. Gut distribution of cecal bacteria at the **(C)** phylum and analysis of differential communities **(D)** at the genus levels. LDA scores **(E)** for differentially abundant genera (LDA score ≥ 2, *p* < 0.05). CON, rabbits fed with the basal diet; YP, rabbits fed with the basal diet and 100 mg/kg yeast peptides; LPS, rabbits challenged by LPS and fed with the basal diet; L-YP, rabbits challenged by LPS and fed with the basal diet and 100 mg/kg yeast peptides (*n* = 4).

Based on the results of taxonomic analysis, we enumerated the species composition and relative abundance of each group at the phylum and genus levels, creating a community histogram for visualization (Figure 8). At the phylum level, the top 10 species in relative abundance were analyzed as dominant phyla. As illustrated in Figure 8A, Firmicutes constituted the most abundant phylum in the cecum microbiota of rabbit (71.17–76.91%), followed by Bacteroidetes (13.65–20.95%) and Verrucomicrobia (3.34–4.24%). In the cecum contents of rabbits, a total of 150 genera were detected, with the top 20 genera in abundance summarized in a bar chart (Figure 8B). The relative abundance of 11 genera exceeded 3%, including UCG-014 (9.10–13.05%), Muribaculaceae (8.91–15.21%), NK4A214 (7.13–11.25%), unclassified Lachnospiraceae (3.72–8.65%), norank Eubacteriaceae (3.61–6.22%), and Clostridia vadinBB60 group (3.35–4.88%).

Figure 8C depicts the abundance distribution of cecum microorganisms at the phylum level. Notably, Proteobacteria emerged as the primary distinctive phylum. Under LPS stress, there was a non-significant increase in Proteobacteria compared to the control group (*p* > 0.05). Conversely, yeast peptide administration reversed this trend, resulting in a significantly lower abundance of Proteobacteria in the L-YP group compared to the LPS group (*p* < 0.05). Additionally, in the YP group, Firmicutes exhibited a non-significant decrease (*p* > 0.05), while Bacteroidetes displayed a non-significant increase (*p* > 0.05) compared to the control group. At the genus level, differences were observed in 12 bacterial genera (Figure 8D). The YP group exhibited a significantly higher abundance of *Butyricimonas* (*p* < 0.05) and increased abundance of *Lactobacillus* (*p* < 0.05), alongside decreased abundance of *Flavobacteriaceae* and *Brevibacterium* (*p* < 0.05) compared to the control group. Conversely, LPS stress led to a significant decrease in the relative abundance of *Subdoligranulum*, *Erysipelotrichaceae*, and *Eubacterium* (*p* < 0.05). In the L-YP group, *Faecalibacterium* showed increased abundance (*p* < 0.05), while the relative abundance of *Rhodospirillales*, *Bilophila*, *Olsenella* and *UCG-002* significantly decreased (*p* < 0.05). To capture additional taxonomic units, LEfSe analysis with LDA (LDA score ≥ 2, *p* < 0.05, Figure 8E) scoring was employed. LEfSe identified 30 divergent taxa through linear analysis. The control group exhibited enrichment in *Erysipelotrichaceae*, *UCG-001*, *UCG-002*, *Oscillospira* and *Brevibacterium*. In the YP group, *Subdoligranulum*, *Butyricimonas*, *Clostridium* and *Bacteroidales* were significantly enriched, including the *rare Chlorella* spp., with *Subdoligranulum* having the highest LDA score. The LPS group displayed higher abundance in the Proteobacteria, *Gammaproteobacteria*, *Burkholderiales* and *Tyzzerella*. In contrast, the L-YP group showed differential enrichment in *Blautia*, *Lactobacillales*, *Odoribacter* and *Alistipe*.

### Correlation analysis

**Figure 9:**
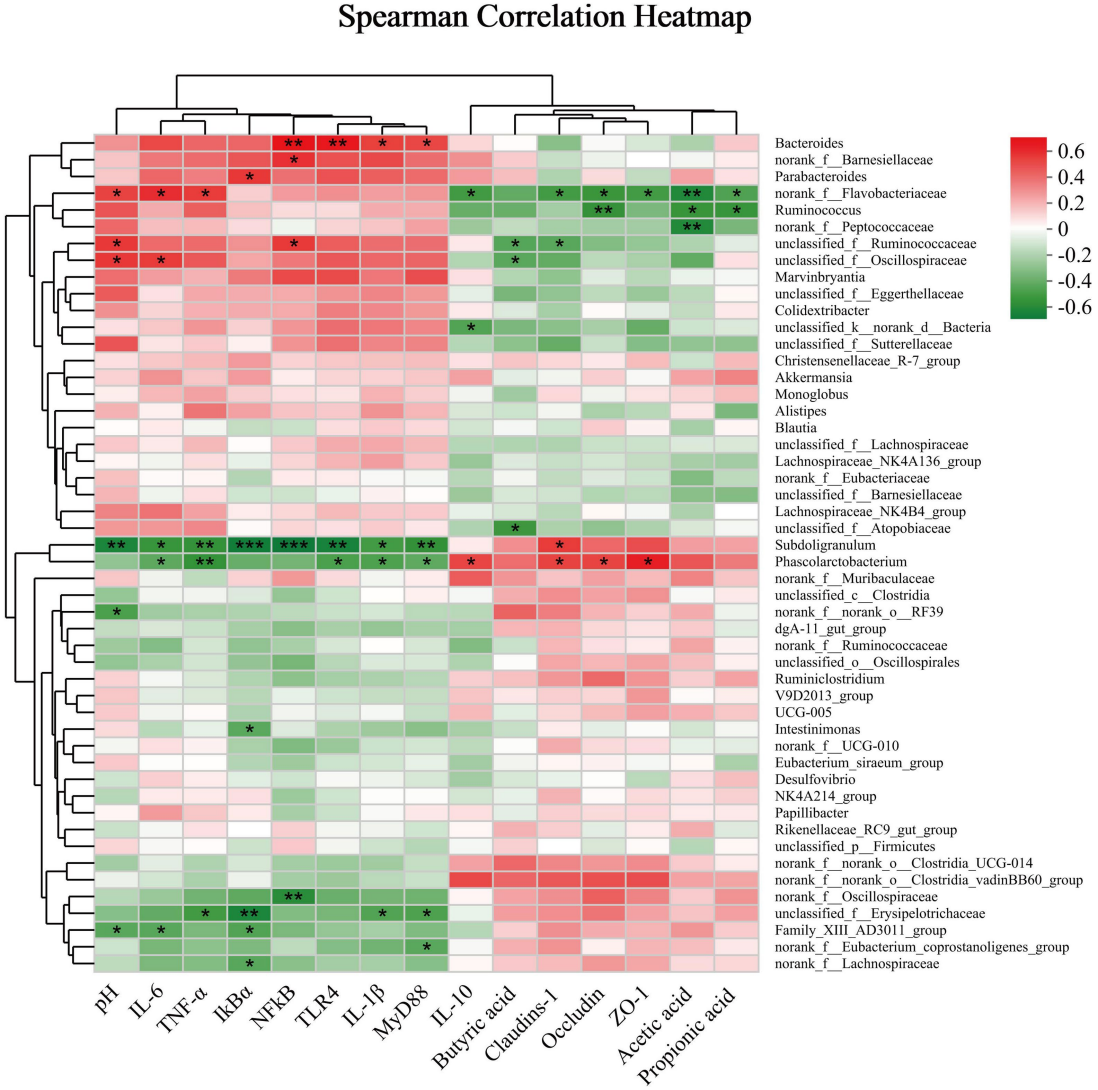
The correlation coefficient between the microbial genus and short-chain fatty acid intestinal barrier parameters. Significance was set at ^*^p < 0.05 and ^**^p < 0.01.

To evaluate the correlation between microbial taxa and environmental variables, correlation heatmap plots were generated to visually represent their relationships. At the genus level (Figure 9), cecum pH exhibited significant positive correlations with the relative abundance of *Flavobacteriaceae*, *Ruminococcaceae*, and *Oscillospiraceae* (*p* < 0.05). Conversely, it showed significant negative correlations with *Subdoligranulum* and *Family XIII AD3011 group* (*p* < 0.05). *Subdoligranulum* demonstrated a highly significant negative correlation with mRNA expression of inflammatory pathway-related factors, including *TNF-α*, *IkBα*, *NF-kB*, *TLR4*, *MyD88*, *IL-6* and *IL-1β* (*p* < 0.05). Moreover, *Family XIII AD3011 group*’s relative abundance was significantly negatively correlated (*p* < 0.05) with *IL-6* and *IkBα* mRNA expression, while *Erysipelotrichaceae*’s relative abundance was significantly negatively correlated (*p* < 0.05) with *TNF-α*, *IkBα*, *IL-1β* and *MyD88* mRNA expression. *IkBα* mRNA expression also exhibited a significant negative correlation (*p* < 0.05) with *unclassified Lachnospira*. Conversely, *IL-6* and *TNF-α* mRNA expression displayed a significant positive correlation with the relative abundance of *Flavobacteriaceae* (*p* < 0.05). *Bacteroides*’ relative abundance exhibited a strong positive correlation with the mRNA expression of *NF-kB* and *TLR4* (*p* < 0.05), as well as a positive correlation with *IL-1β* and *MyD88* mRNA expression (*p* < 0.05). *Phascolarctobacterium*’s relative abundance was significantly negatively correlated (*p* < 0.05) with mRNA expression of *IL-6*, *TNF-α*, *TLR4*, *IL-1β* and *MyD88*. Conversely, *Phascolarctobacterium* demonstrated a significant positive correlation (*p* < 0.05) with intestinal barrier-related genes (*Occludin*, *Claudins-1* and *ZO-1*) and mRNA expression of *IL-10*. Furthermore, mRNA expression of *Occludin*, *Claudins-1*, *ZO-1* and *IL-10* was significantly negatively correlated with the relative abundance of *Flavobacteriaceae* (*p* < 0.05). Occludin exhibited a significant negative correlation with the relative abundance of *Ruminococcus* (*p* < 0.05). All SCFA contents in the cecum were significantly negatively correlated (*p* < 0.05) with *Flavobacteriaceae*. Specifically, acetic acid content showed a significant negative correlation (*p* < 0.05) with the relative abundance of *Peptococcaceae*, propionic acid displayed a significant negative correlation (*p* < 0.05) with the relative abundance of *Ruminococcus*, and butyric acid was significantly negatively correlated (*p* < 0.05) with the relative abundance of *Peptococcaceae*.

## Discussion

Yeast peptide is a kind of antibacterial peptide produced by fermentation of *Saccharomyces cerevisiae*, which can improve the intestinal environment, regulate the balance of intestinal flora, improve the immune ability of animals, and thus improve the growth performance and health level of animals (16). In this investigation, we examined the impact of incorporating dietary yeast peptides on intestinal barrier and inflammation in rabbit subjected to LPS challenge. The intestinal function extends beyond nutrient digestion and absorption, serving as a crucial barrier against harmful substances (17). Compromised integrity of this barrier facilitates the passage of bacterial components, metabolites, and harmful substances, leading to systemic inflammation (18). Serum DLA and DAO serve as indicators of intestinal permeability and are positively correlated with it. In our study, following the LPS challenge, animals exhibited a significant elevation in serum DLA levels and DAO activity (19). Interestingly, yeast peptides mitigated this effect, attenuating the LPS-induced rise in DLA and DAO levels and preserving intestinal barrier integrity. Numerous animal experiments have shown that LPS induces severe intestinal damage, including villi atrophy, mucosal detachment, and deepening of crypts (20, 21). In contrast, yeast peptides shield intestinal villi from damage, maintaining the normal structure and morphology of the intestine (16). In our study, we found that exposure to LPS led to significant congestion in the rabbit intestine, along with reduced villi height, VCR, and cecal thickness in the jejunum, indicating acute damage. However, the addition of yeast peptides helped alleviate the adverse effects of LPS on the intestine. Additionally, we analyzed tight junctions between intestinal epithelial cells. ZO-1, Occludin, and Claudins represent the three pivotal tight junction proteins determining intestinal permeability (22). In our study, we found that LPS treatment significantly reduced mRNA expression of jejunal ZO-1, Occludin, and Claudins-1 in rabbits, leading to increased intestinal permeability and dysfunction of the intestinal barrier. This aligns with prior studies indicating that antimicrobial peptides enhance intestinal integrity and selective permeability during *E. coli* attacks (23–25). Conversely, yeast peptides had a notable effect on tight junction proteins. Although the specific regulatory mechanism of yeast peptides on tight junction proteins remains unclear, existing studies suggest two potential pathways: firstly, activation of regulatory proteins in the intestine, such as the Rho family, increasing the expression of tight junction proteins; and secondly, reduction of damage to tight junction proteins through the inhibition of pathogenic bacteria, ensuring the integrity of the intestinal barrier (26, 27).

The regulation of intestinal inflammation involves cytokines, and an excessive production of inflammatory cytokines (e.g., TNF-α, IL-1β, and IL-8, etc.) constitutes a prominent characteristic of LPS-induced intestinal injury (28). Prior research has demonstrated the efficacy of diminishing pro-inflammatory factor levels in alleviating intestinal inflammation and countering the disruption of the intestinal barrier function caused by pathogenic bacteria and harmful substances (29). Another investigation reported that the antimicrobial peptide LFP-20 prevented LPS-induced damage to colonic epithelial tissue, infiltration of macrophages or leukocytes, and elevated levels of TNF-α, IL-6, and IFN-γ (30). Consistent findings were observed in our study, where yeast peptides decreased the concentrations of TNF-α, IL-1β, IL-6, and IFN-γ in the intestines of LPS-induced rabbits. A significant finding is that yeast peptides mitigate the LPS-induced downregulation of IL-10. IL-10 plays a crucial role in inhibiting the synthesis and bioactivity of inflammatory factors, suppressing antigen presentation response, and reducing the release of inflammatory cytokines. Hence, the inhibitory impact of yeast peptides on inflammation may also be realized through the modulation of anti-inflammatory factors.

To delve deeper into the molecular mechanisms underlying the attenuation of intestinal inflammation by yeast peptides, we investigated the TLR4/NF-κB signaling pathway. During inflammation, LPS challenge activate the TLR4 signaling pathway and regulate downstream proteins such as MyD88 and NF-κB to spread inflammation (31, 32). In addition, it can also directly induce phosphorylation of IκB-α, leading to increased NF-κB phosphorylation and induced high production of downstream pro-inflammatory factors such as TNF-α and IL-1β (33, 34). Study has demonstrated the capacity of the antimicrobial peptide to mitigate LPS-induced macrophage inflammation by suppressing the NF-κB signaling pathway (35). In our investigation, yeast peptide supplementation counteracted the LPS-induced elevation in mRNA expression levels of *TNF-α*, *MyD88*, *NF-κB*, *IL-1β* and *IL-6*, while concurrently preventing the decrease in mRNA expression levels of *IκB-α* and *IL-10*. This suggests that yeast peptides may modulate inflammatory signaling by regulating pivotal central factors like NF-κB and IκB-α, contributing to the suppression of inflammation.

Short-chain fatty acids (SCFA), produced through the fermentation of dietary fiber by anaerobic microorganisms in the intestines, not only underscore the close association with gut health but also serve a crucial role in the body as both an energy substrate and a signaling mediator (36, 37). Research indicates that SCFA not only provides energy to intestinal epithelial cells and certain bacteria but also enhances the expression of tight junction proteins (38, 39). In our current investigation, the introduction of yeast peptides led to an elevation in SCFA concentration (acetate, propionate, and butyrate) in intestines subjected to LPS. This observation supports the notion that yeast peptides mitigated LPS-induced intestinal damage and suppressed inflammatory responses, potentially attributed to the heightened levels of SCFA.

The pivotal regulatory role of the gut microbiota in host health and disease is widely acknowledged (40). A well-balanced microbiota contributes to the regulation of the body’s immunity and metabolism by producing host-beneficial small-molecule metabolites and mending the integrity of the intestinal mucosal barrier (41, 42). Conversely, dysregulation of the gut microbiota may compromise normal immune function and escalate into severe metabolic disorders and intestinal inflammation (43, 44). In our current investigation, Firmicutes, Bacteroidetes, and Verrucomicrobia emerged as the predominant bacterial taxa in the cecum microbiota at the phylum level, aligning with previous research findings (45). A more detailed examination at the phylum level disclosed a noticeable difference between groups, specifically the elevation of Proteobacteria due to LPS attack. Remarkably, yeast peptides counteracted the adverse effects induced by LPS. Notably, increased Proteobacteria is identified as a key feature in the development of endotoxemia and metabolic disorders (46, 47). At the genus level, the gut microbiota significantly augmented by yeast peptides in this study included *Butyricimonas*, *Lactobacillus*, and *Faecalibacterium*. *Butyricimonas* and *Lactobacillus*, recognized as beneficial bacteria in the intestinal tract, demonstrate anti-inflammatory properties by producing SCFA (48). An investigation demonstrated that both live and heat-inactivated *Butyricimonas virosa* mitigated adverse effects such as weight loss, hyperglycemia, and steatosis—in diseased mice by activating Glucagon-like peptide 1 receptors (GLP-1R) in the liver within an obese mouse model (49). In alignment with our results, Zhu et al. observed that the supplementation of antimicrobial peptides (MPX) to broiler diets increased the relative abundance of beneficial bacteria such as *Lactobacillus*, *Lactococcus* and *Parabacteroides* in the cecum, concurrently reducing pathogenic bacterial content (50). This dietary intervention alleviated inflammation of the intestinal mucosa and enhanced barrier function. Fan et al. (51) observed that an augmented presence of *Lactobacillus* in the intestine effectively mitigated collagenous arthritis (CIA) in female Wistar rats. This reduction was achieved through the down-regulation of various pathways, including decreased pro-inflammatory cytokine content, SFCA production, and modulation of Th1/Th17. *Faecalibacterium*, a prominent bacterial species in the colons of healthy adults, is often considered pivotal for human intestinal health (52). Its positive effects can be attributed to its role as a butyrate-producing bacterium in the intestinal tract and its anti-inflammatory functions (53). Tang et al. reported that *Faecalibacterium* attenuates inflammation by inhibiting NF-κB activation and reducing the secretion of pro-inflammatory factors (36). Furthermore, yeast peptides led to a decrease in the relative abundance of *Flavobacteriaceae*, *Brevibacterium*, *Rhodospirillales*, *Bilophila* and *Olsenella*. *Flavobacterium* and *Rhodospirillales*, both Gram-stain-negative bacteria, are potentially pathogenic; the former may cause pneumonia, meningitis, and sepsis when the body’s immunity is compromised, while the latter has been linked to reliance on the breakdown of cecal organisms (fatty acids) for growth, though its categorization as a disease-causing factor remains uncertain (54, 55). These findings collectively suggest that the dietary supplementation of yeast peptides may alleviate LPS-induced intestinal inflammation by fostering the colonization of beneficial intestinal bacteria and inhibiting pathogenic counterparts. LDA analysis yielded consistent outcomes, revealing that LPS induced a substantial enrichment of pathogenic bacteria, particularly Proteobacteria, in the cecum, resulting in a disruption of the intestinal flora. In stark contrast, the yeast peptide exhibited a significant enrichment of beneficial bacteria. The YP group displayed a more robust and diverse assembly of beneficial bacteria. Notably, the significantly enriched bacterial colonies (*Blautia*, *Lactobacillales*, *Odoribacter*, and *Alistipe*) appeared to play a pivotal role in this defensive mechanism. A study involving human females demonstrated that, under chronic stress conditions, decreased levels of *Blautia* and its metabolite, acetic acid, in the intestinal tract foster the development of breast cancer (56). *Lactobacillales* are renowned for fermenting sugars to produce lactic acid, known for its immune-enhancing properties and physiological activities, including promoting energy metabolism, anti-inflammatory effects, and reducing the risk of cancer (57, 58). *Odoribacter*, with its capability to produce SCFA, demonstrated a negative correlation with inflammatory bowel disease, portraying its role as a beneficial commensal interacting positively with the host. Additionally, *Alistipe*, associated with potential protective effects against various diseases (liver fibrosis, enteritis, immune, and cardiovascular diseases) was identified (59). These results highlight the yeast peptides’ significant and positive regulation of the intestinal flora.

Correlation analysis underscored the significant negative correlation of *Subdoligranulum* with almost all inflammatory factors. In contrast, it exhibited a positive correlation with the mRNA expression of intestinal tight junction proteins. This underscores the crucial role of *Subdoligranulum* as one of the key flora contributing to yeast peptides’ effectiveness in improving intestinal inflammation and barrier integrity. It has been demonstrated that *Subdoligranulum* is prevalent in healthy populations and nearly absent in obese and diabetic cohorts (60). Consistent with our findings, Leclercq et al. illustrated a positive correlation between *Subdoligranulum* abundance and intestinal integrity (61). *Phascolarctobacterium* exhibited a positive correlation with mRNA expression of intestinal tight junction proteins and SCFA, along with a negative correlation with inflammatory factors, suggesting that yeast peptides enhance the intestinal barrier and mitigate inflammation by modulating SCFA and intestinal microbiota composition. Conversely, as a conditionally pathogenic bacterium, *Bacteroides* exhibited a high correlation with inflammatory factors, particularly NF-κB and TLR4. On the contrary, *Lachnospiraceae*, which is negatively correlated with inflammatory factors, is an indisputably beneficial bacterium that plays a crucial role in fermenting dietary fibers to produce acetic and butyric acids, consequently supplying energy to the host (62). Consequently, the regulation by yeast peptides on intestinal microbiota may be a key factor in improving the barrier function of rabbits’ intestinal mucosa. The underlying mechanism needs to be further studied.

## Conclusion

In conclusion, the incorporation of yeast peptides into the rabbit diet has exhibited a significant enhancement in intestinal health. The underlying mechanisms of this improvement may be associated with the modulation of the Toll-like receptor signaling pathway and regulation of the gut microbiota. Our findings may serve as a basis for better understanding the beneficial effects of yeast peptides in the field of animal nutrition, and to maximize the protective effects of dietary yeast peptides supplementation against intestinal injury in animals.

## Data availability statement

The datasets presented in this study can be found in online repositories. The names of the repository/repositories and accession number(s) can be found in the article/supplementary material.

## Ethics statement

The animal studies were approved by Animal Care and Use Committee of Hebei Agriculture University (Baoding, China). The studies were conducted in accordance with the local legislation and institutional requirements. Written informed consent was obtained from the owners for the participation of their animals in this study.

## Author contributions

JF: Investigation, Methodology, Writing – original draft, Writing – review & editing. CL: Software, Validation, Writing – review & editing. WH: Investigation, Writing – review & editing. FW: Investigation, Writing – review & editing. HF: Investigation, Writing – review & editing. DF: Investigation, Writing – review & editing. YL: Investigation, Writing – review & editing. ZG: Investigation, Writing – review & editing. YW: Investigation, Writing – review & editing. SC: Conceptualization, Methodology, Writing – review & editing. BC: Methodology, Resources, Writing – review & editing.

## Glossary

CIA: Collagenous arthritis
DAO: Diamine oxidase
DLA: D-lactic acid
GLP-1R: Glucagon-like peptide 1 receptors
IL-10: Interleukin-10
IL-1β: Interleukin-1 beta
IL-6: Interleukin-6
INF-γ: Interferon-gamma
IκBα: Inhibitor of nuclear factor kappaB
LEfSe: Linear Discriminant Analysis Effect Size
LPS: Lipopolysaccharide
MyD88: Myeloiddifferentiationfactor 88
NF-κB: Nuclear factor kappa-B
OTU: Operational Taxonomic Unit
PCoA: Principal Coordinate Analysis
SCFA: Short-chain fatty acid
SIgA: Simplified improved Gaussian approximation
TLR4: Toll-like receptors 4
TNF-α: Tumor necrosis factor-alpha
UC: Ulcerative colitis
VCR: Ratio of villus height to crypt depth
ZO-1: Zonula occludens-1
